# The role of metabolic reprogramming in liver cancer and its clinical perspectives

**DOI:** 10.3389/fonc.2024.1454161

**Published:** 2024-11-14

**Authors:** Mengxiao Lu, Yingjie Wu, MinMing Xia, Yixin Zhang

**Affiliations:** Department of Gastrointestinal Minimally Invasive Surgery, The Affiliated People’s Hospital of Ningbo University, Ningbo, China

**Keywords:** liver cancer, hepatocellular carcinoma, metabolic dysfunction-associated steatotic liver disease, metabolic reprogramming, Warburg effect, lipid metabolism, amino acid metabolism

## Abstract

Primary liver cancer (PLC), which includes hepatocellular carcinoma (HCC) and intrahepatic cholangiocarcinoma (iCCA), remains a leading cause of cancer-related death worldwide. Chronic liver diseases, such as hepatitis B and C infections and metabolic dysfunction-associated steatotic liver disease (MASLD), are key risk factors for PLC. Metabolic reprogramming, a defining feature of cancer, enables liver cancer cells to adapt to the demands of rapid proliferation and the challenging tumor microenvironment (TME). This manuscript examines the pivotal role of metabolic reprogramming in PLC, with an emphasis on the alterations in glucose, lipid, and amino acid metabolism that drive tumor progression. The Warburg effect, marked by increased glycolysis, facilitates rapid energy production and biosynthesis of cellular components in HCC. Changes in lipid metabolism, including elevated *de novo* fatty acid synthesis and lipid oxidation, support membrane formation and energy storage essential for cancer cell survival. Amino acid metabolism, particularly glutamine utilization, supplies critical carbon and nitrogen for nucleotide synthesis and maintains redox homeostasis. These metabolic adaptations not only enhance tumor growth and invasion but also reshape the TME, promoting immune escape. Targeting these metabolic pathways presents promising therapeutic opportunities for PLC. This review underscores the interaction between metabolic reprogramming and tumor immunity, suggesting potential metabolic targets for innovative therapeutic strategies. A comprehensive understanding of PLC’s intricate metabolic landscape may lead to more effective treatments and better patient outcomes. Integrating metabolomics, genomics, and proteomics in future research will be vital for identifying precise therapeutic targets and advancing personalized therapies for liver cancer.

## 1 Introduction

Primary liver cancer (PLC), encompassing hepatocellular carcinoma (HCC) and intrahepatic cholangiocarcinoma (iCCA), is among the leading causes of cancer-related deaths globally. In 2022, liver cancer accounted for over 750,000 fatalities worldwide, making it the third most common cause of cancer mortality, following lung and colorectal cancers, and the sixth most diagnosed malignancy. It ranks as the second deadliest cancer in men, with incidence and mortality rates in men being two to three times higher than in women across most regions (1). PLC is predominantly linked to chronic liver conditions, including hepatitis B and C infections and metabolic dysfunction-associated steatotic liver disease (MASLD, also referred to as non-alcoholic fatty liver disease, NAFLD) (2, 3). The prognosis for liver cancer remains poor, especially in advanced stages, highlighting the need for a comprehensive understanding of PLC pathogenesis to inform the development of new treatment strategies.

Metabolic reprogramming is a key process by which tumor cells sustain rapid proliferation and evade immune surveillance. Unlike normal cells, cancer cells undergo significant metabolic adaptations to satisfy their heightened requirements for energy, biosynthetic precursors, and survival signals. These alterations span various pathways, including enhanced glycolysis, increased fatty acid synthesis, amino acid metabolism, and nucleotide production (3). Metabolic reprogramming not only facilitates tumor cell growth and survival but also significantly influences the immune modulation within the tumor microenvironment (TME).

This review examines the impact of metabolic reprogramming on liver cancer initiation and progression, with a focus on how shifts in metabolic pathways drive tumor growth, invasion, and metastasis. Additionally, it explores the interaction between metabolic alterations and tumor immunity, as well as how targeting these pathways can modulate the immune landscape of the TME. These insights lay the groundwork for developing innovative therapeutic strategies for PLC. By characterizing the metabolic features of PLC and evaluating metabolism-based therapeutic potentials, this review aims to contribute to the advancement of more effective treatment options for patients with liver cancer.

## 2 Overview of metabolic reprogramming in cancer

**Figure 1:**
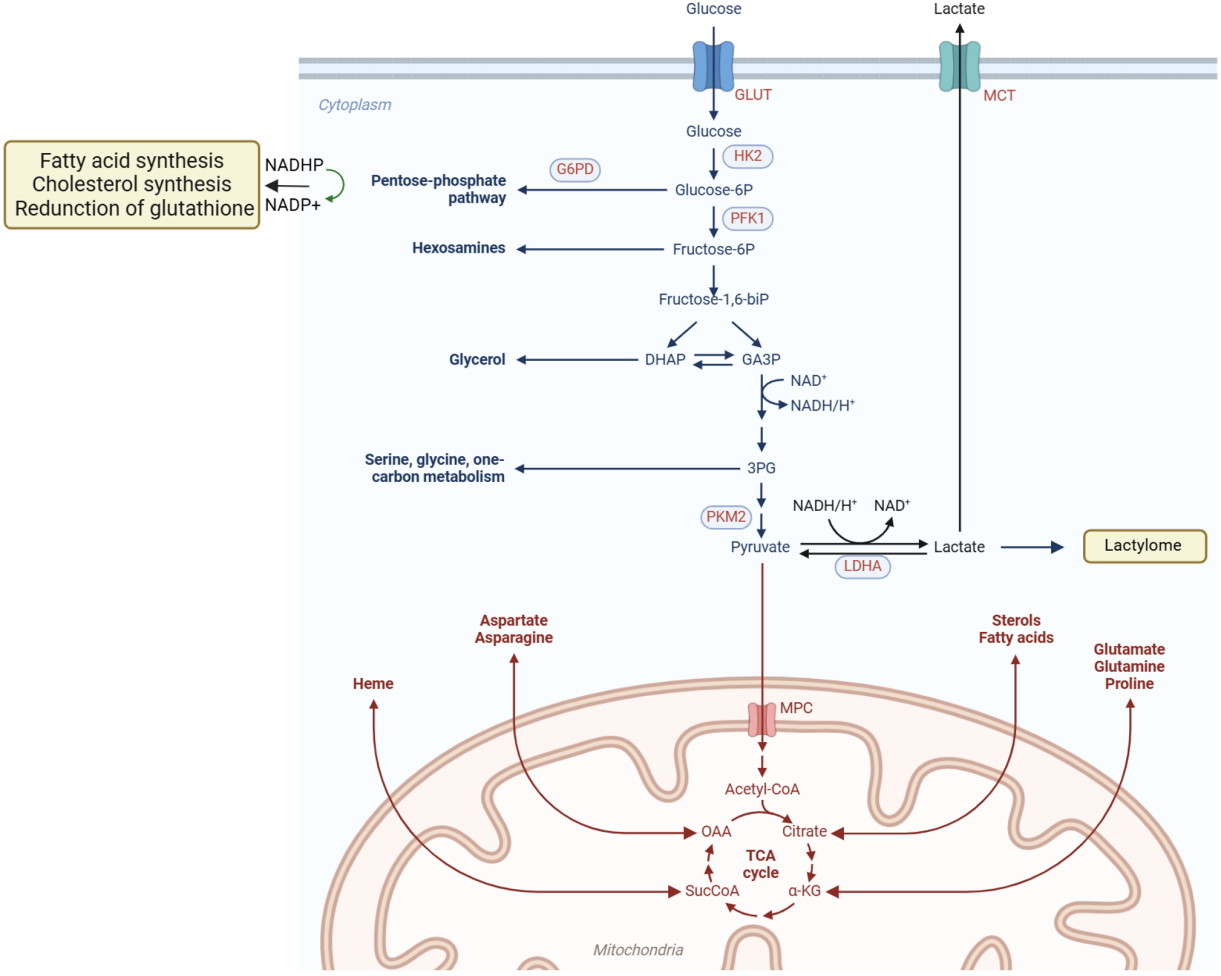
Metabolic reprogramming of tumor cells. Tumor cells actively acquire glucose and other nutrients from the tumor microenvironment, and key metabolic pathways, such as the PPP and lactic acid fermentation, become hyperactivated. Additionally, mitochondrial metabolic reprogramming allows lipids and amino acids to be metabolized through the TCA cycle, generating abundant small-molecule products. These products provide essential substrates and energy to support cell proliferation, migration, invasion, and other malignant behaviors. Metabolic reprogramming in tumor cells encompasses diverse pathways, including glucose, lipid, and amino acid metabolism, enabling them to meet the demands of rapid growth and survival in hostile environments.

Tumor cells undergo metabolic reprogramming to meet the demands of rapid proliferation, involving substantial alterations in glucose, lipid, and amino acid metabolism, among other pathways (**Figure 1**), which are intricately linked to the tumor immune microenvironment (4).

### 2.1 Warburg effect

As early as the 1920s, Otto Warburg observed that cancer cells, even under oxygen-rich conditions, preferentially consume large amounts of glucose and convert it to lactate through glycolysis—a phenomenon termed the Warburg effect (5, 6). This metabolic adaptation not only provides a rapid energy source but also generates essential precursors for biosynthetic processes, such as ribose-5-phosphate and NADPH, which are essential for maintaining the anabolic state and redox balance required for tumor cell growth and survival.

The mechanisms underlying the Warburg effect are multifaceted. First, tumor cells often exhibit mitochondrial dysfunction due to the limited repair capacity of mitochondrial DNA, which lacks introns and protective histones, coupled with a high mutation rate, leading to impaired mitochondrial function (7, 8). Second, dysregulation of oncogenic pathways and tumor suppressor genes, such as RAS, PI3K/AKT, and mutated P53, shifts cellular energy metabolism toward glycolysis, making cancer cells increasingly glycolysis-dependent (9–11). Third, the rapid proliferation of tumor cells generates a hypoxic internal microenvironment, which stabilizes hypoxia-inducible factor 1 alpha (HIF-1α), further enhancing glycolytic activity and inhibiting oxidative phosphorylation (OXPHOS) under low oxygen conditions (12). Despite evidence that the electron transport chain (ETC) and OXPHOS are still involved in tumorigenesis, increased aerobic glycolysis is a consistent finding across various cancers, reinforcing the Warburg effect as a central concept in tumor metabolism. This highlights the intricate interplay between genetic mutations, metabolic reprogramming, and the tumor microenvironment (TME) in promoting cancer cell proliferation and survival, offering insights into potential therapeutic strategies targeting cancer metabolism.

### 2.2 Lipid metabolism

Tumor cells also upregulate *de novo* fatty acid synthesis to support membrane biosynthesis and energy storage by increasing the expression of key enzymes such as fatty acid synthase (FASN) and acetyl-CoA carboxylase (ACC) (13). This pathway is critical for rapidly dividing cancer cells, which require substantial lipid quantities for new membrane formation. FASN catalyzes the synthesis of palmitate, a fundamental saturated fatty acid, while ACC provides malonyl-CoA for fatty acid chain elongation. The upregulation of these enzymes enables tumor cells to efficiently produce the lipids necessary for sustained growth and division.

### 2.3 Amino acid metabolism

Amino acid metabolism, especially glutamine metabolism, is pivotal for tumor cell proliferation, migration, and invasion. Glutamine serves as a key nutrient for many cancers, providing both carbon and nitrogen that support multiple cellular functions. It acts as a precursor for the synthesis of purines and pyrimidines, which are essential for DNA and RNA production, thereby enabling the rapid cell division characteristic of cancer. Additionally, glutamine plays a critical role in maintaining redox homeostasis within tumor cells by contributing to the production of glutathione, a major antioxidant that shields cancer cells from oxidative stress and damage, thereby enhancing their survival (14). Given these dependencies, certain cancers exhibit “glutamine addiction,” heavily relying on glutamine to sustain their metabolic processes (15). This dependency creates a potential therapeutic opportunity, as targeting glutamine metabolism could selectively affect cancer cells while sparing normal cells that have a lower reliance on this amino acid. Thus, a deep understanding of glutamine’s role in cancer metabolism could facilitate the development of therapies aimed at disrupting these pathways to inhibit tumor growth and progression.

### 2.4 Other metabolisms

Tumor cells also reprogram other metabolic pathways, including the pentose phosphate pathway (PPP), the one-carbon cycle, and nucleotide metabolism, to fulfill their biosynthetic and energetic demands (16). These metabolic adaptations are essential for supporting the rapid proliferation of cancer cells and can significantly influence the stability of the TME. For instance, glucose-6-phosphate dehydrogenase (G6PDH), a key regulatory enzyme in the PPP, is upregulated by oncogenic signals such as ATM, PI3K/AKT, RAS, and SRC (16, 17). The upregulation of G6PDH represents a key adaptation, as it activates the pentose phosphate pathway (PPP), supplying cancer cells with ample pentose phosphate and NADPH. Pentose phosphate is vital for nucleic acid synthesis, enabling the rapid DNA replication and repair necessary for tumor growth (16). NADPH is equally important, as it supports fatty acid synthesis and helps maintain cellular redox balance, shielding cells from oxidative stress. By activating the PPP, tumor cells secure a continuous supply of these essential molecules, thus fulfilling their anabolic requirements and promoting survival and proliferation. These metabolic changes do not occur in isolation but are intricately connected with other pathways, such as the one-carbon cycle and nucleotide metabolism, forming a comprehensive network that meets the high biosynthetic and energy needs of cancer cells (17). Understanding the interactions among these pathways offers valuable insights into potential therapeutic targets, as disrupting these networks could compromise the metabolic adaptability of cancer cells and inhibit tumor growth.

### 2.5 Metabolism and TME

The metabolic activity of tumor cells is intricately linked to immune cell function within the TME. Metabolites such as lactic acid can modulate immune cell behavior and facilitate tumor immune evasion (18). Competition for metabolic resources between tumor cells and immune cells influences immune efficacy and impairs tumor immune surveillance (18). Tumor cell metabolic reprogramming not only sustains their growth and survival but also drives tumor progression and metastasis by reshaping the TME and modulating immune responses. These metabolic adaptations present diverse therapeutic targets for novel strategies aimed at exploiting the metabolic vulnerabilities of tumor cells. Further research is needed to unravel the complexities of tumor metabolism and develop targeted metabolic therapies to improve cancer patient outcomes (19).

## 3 Role and related mechanisms of metabolic reprogramming in PLC

**Figure 2:**
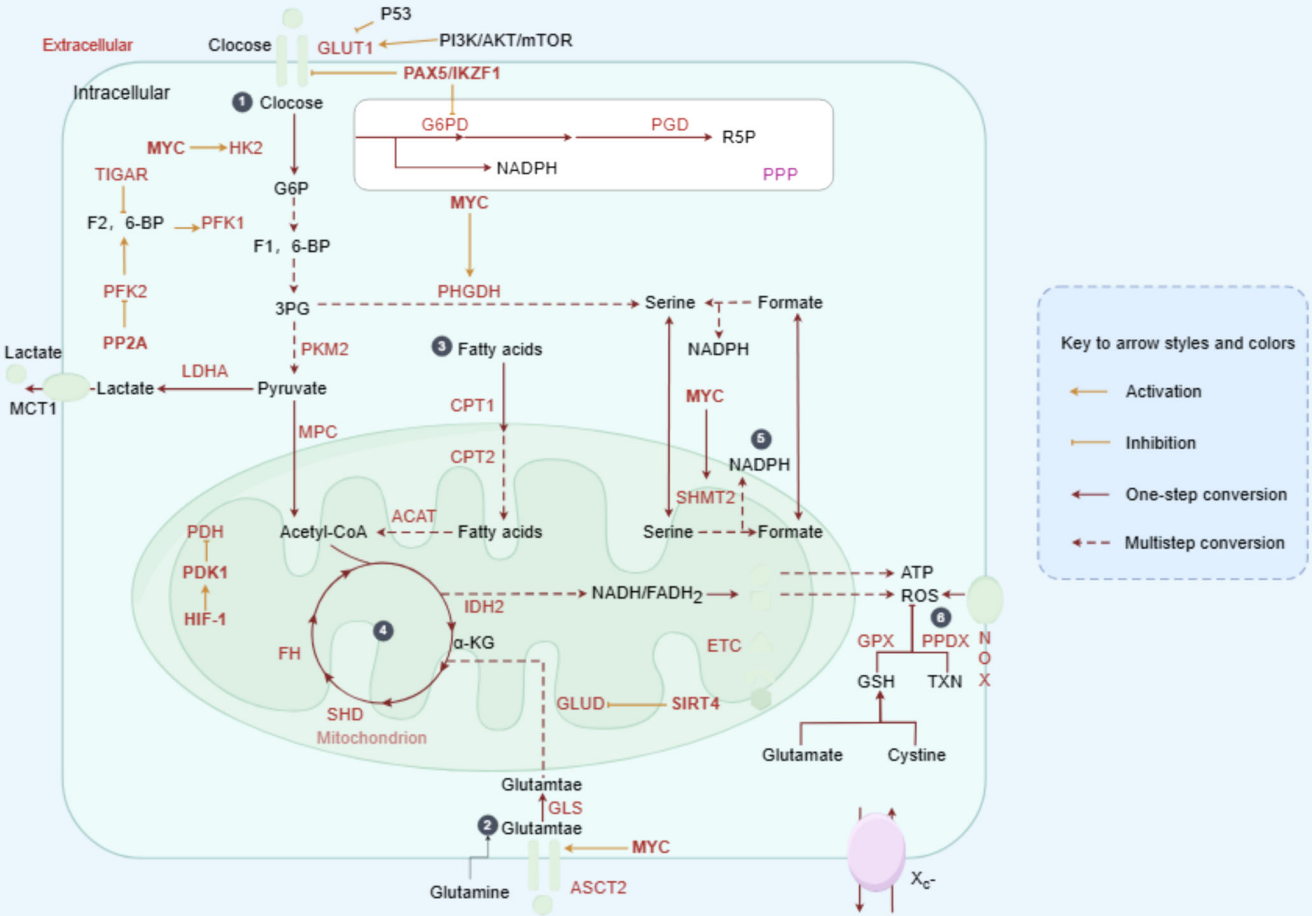
Metabolic reprogramming drives HCC progression. In HCC, oncogenic factors stimulate the abnormal activation of various metabolic enzymes. Glucose is taken up by cancer cells and converted into pyruvate via glycolysis. While the majority of pyruvate is fermented to lactate, a smaller portion is transported into mitochondria and converted to acetyl-CoA, fueling the TCA cycle and OXPHOS. Cancer cells also show enhanced lipid uptake and synthesis, storing lipids or utilizing them for beta-oxidation in mitochondria. Amino acids, such as glutamine, replenish the TCA cycle through conversion to alpha-ketoglutarate. The PPP, a branch of glycolysis, produces ribulose-5-phosphate, NADPH, and precursors for the biosynthesis of cholesterol, fatty acids, and nucleotides, as well as reducing glutathione. Additionally, the serine synthesis pathway branches from glycolysis, with SHMT converting serine to glycine, generating THF and 5,10-methylene-THF for DNA and RNA synthesis, or conversion to the methyl-donor S-adenosylmethionine.

As the primary organ regulating metabolism, the liver is closely associated with the pathogenesis of liver cancer, with metabolic disorders playing a significant role in its development. Major risk factors for liver cancer include HBV/HCV infection, alcohol consumption, obesity, and metabolic dysfunction-associated steatotic liver disease (MASLD). Additional risk factors for iCCA include aflatoxin exposure, liver fluke infection, bile duct cysts, and primary sclerosing cholangitis (PSC) (20). These factors may contribute to PLC through metabolic reprogramming mechanisms (21). Elucidating the metabolic alterations in PLC is therefore critical for identifying pathogenic mechanisms and therapeutic targets. The following section will focus on HCC to illustrate the central role of metabolic reprogramming in PLC (**Figure 2**).

### 3.1 Glucose metabolism in HCC

Systematic analyses of metabolic gene expression and untargeted metabolomic profiling have identified aerobic glycolysis, lipid metabolism, and amino acid metabolism as the predominant metabolic alterations in HCC tissues (22). These findings underscore the significance of the Warburg effect as a central metabolic adaptation in certain HCC tumors. Elevated expression of glucose transporter 1 (GLUT1) is commonly observed in HCC and is associated with poorer patient prognosis (23, 24), largely due to its correlation with TP53 mutations and activation of the PI3K/AKT/mTOR signaling pathway (25). Hexokinase 2 (HK2), which catalyzes the conversion of glucose to glucose-6-phosphate (G6P) in the initial step of glycolysis, is also upregulated in HCC, with higher expression linked to reduced overall survival (26–28). Multiomic analysis of 65 human HCC organoids further delineated two distinct metabolic subtypes, enhancing the understanding of metabolic diversity in HCC tissues. Notably, glucose-6-phosphate dehydrogenase (G6PD) emerged as a potential therapeutic target, particularly in drug metabolism-enriched pathways (29).

A comprehensive meta-analysis of 521 HCC samples and 2,761 metabolic genes revealed 284 upregulated and 350 downregulated metabolic genes in HCC cohorts (30). Upregulated genes were associated with glycolysis, PPP, nucleotide biosynthesis, TCA, OXPHOS, proton transport, and membrane lipid and glycan metabolism. In contrast, downregulated genes were primarily involved in xenobiotic, fatty acid, and amino acid metabolism. Given the hypoxic and nutrient-poor environment encountered by cancer cells, metabolic reprogramming is necessary to satisfy energy and biosynthetic demands. Retrospective studies have consistently shown that elevated serum LDH levels correlate with poor prognosis in HCC and iCCA following radical resection or standard therapies (31, 32). Under hypoxic conditions, enhanced glycolysis in liver cancer cells activates lactate dehydrogenase A (LDHA), leading to increased lactate production (33). Lactate accumulation not only acidifies the microenvironment but also induces lactoylation of adenylate kinase 2 (AK2) at lysine 28, impairing its kinase activity and disrupting energy homeostasis, thereby promoting tumor proliferation, invasion, and metastasis (34). Notably, the triterpenoid compound demethylzeylasteral (DML) has been shown to inhibit tumorigenesis in liver cancer stem cells (LCSCs) by interfering with lactylation at histone marks H3K9la and H3K56la, linked to metabolic stress responses (35). Hypoxia also activates macropinocytosis through the HIF-1α/EH domain-containing protein 2 (EHD2) pathway to facilitate nutrient uptake in several HCC cell lines and mouse models (36). Additionally, other glucose metabolism pathways, including the PPP and hexosamine biosynthesis pathway (HBP), exhibit heightened activity in HCC tissues (37–39).

### 3.2 Lipid metabolism in HCC

A study employing single-cell RNA sequencing (scRNA-seq), lipidomics, metabolomics, and proteomic analysis in 41 patients with HCC classified the tumors into three metabolic subtypes (F1, F2, and F3) based on fatty acid oxidation (FAO) activity. Subtype-specific therapeutic strategies were explored by analyzing clinical, mutational, epigenetic, metabolic, and immunological characteristics. The F1 subtype, with the lowest FAO activity, responded favorably to treatments such as YM-155, Alisertib, sorafenib, anti-PD1, anti-PD-L1, and the combination of atezolizumab and bevacizumab (T+A). In contrast, the F3 subtype, characterized by the highest FAO activity, showed responsiveness to transarterial chemoembolization (TACE), while F2 represented an intermediate state between F1 and F3 (40). In high-fat diet (HFD)-induced HCC or steatohepatitis, the FAO pathway is typically downregulated, potentially protecting HCC cells from lipotoxicity (41). Concurrently, increased expression of fatty acid synthase (FASN) and acetyl-CoA carboxylase (ACC), key enzymes involved in *de novo* fatty acid synthesis, is observed in HCC, with elevated lipogenesis often linked to poor outcomes in advanced HCC cases (42–45). Interestingly, liver-specific ACC knockout in diethylnitrosamine (DEN)-induced HCC mouse models revealed that inhibiting *de novo* lipogenesis could accelerate tumor progression by activating the antioxidant defense system, underscoring the metabolic adaptability of tumors and the necessity for further investigation into its role in HCC (46). Lipid metabolic pathways, including FASN and stearoyl-CoA desaturase (SCD) signaling, also play pivotal roles in maintaining cancer stem cell (CSC) populations, thereby driving metastasis and drug resistance in HCC (47). Moreover, a high-cholesterol diet disrupts metabolism and calcium signaling, leading to HCC development in mouse models (48–50). Integrated proteomic and phosphoproteomic studies have demonstrated that targeting sterol O-acyltransferase 1 (SOAT1) to lower plasma membrane cholesterol content offers a promising therapeutic approach for early-stage HCC, as validated in patient-derived xenograft mouse models (51). Conversely, elevated serum cholesterol levels have been associated with improved outcomes in patients with HCC by inhibiting tumor metastasis, suggesting that cholesterol distribution and homeostasis play significant roles in the onset and progression of HCC (52).

### 3.3 Amino acid metabolism in HCC

Studies have consistently demonstrated that amino acid metabolism is upregulated in liver tumors compared to non-tumor tissues (20, 53, 54). Glutamine, the most abundant amino acid in human blood, serves as a critical carbon source for the TCA cycle and provides essential nitrogen for the synthesis of purine and pyrimidine nucleotides, which are necessary for DNA and RNA production (55). In HCC models, glutamine addiction, particularly in tumors overexpressing glutamine synthetase (GS), supports mTOR-dependent cell proliferation and survival (46). Additionally, tumor tissues exhibit an increased glutamate-to-proline biosynthetic flux, which facilitates HCC cell growth and tumor expansion (56). Urea cycle dysregulation is a characteristic metabolic alteration in human HCC, where tumor cells redirect urea metabolism away from arginine synthesis toward pyrimidine biosynthesis. Consequently, HCC cells become reliant on exogenous arginine, and arginine deprivation triggers a GCN2 kinase-mediated stress response, leading to cell cycle arrest and reversible quiescence. Inhibition of GCN2 disrupts these protective responses, inducing senescence and sensitizing HCC cells to apoptosis *via* senolytic agents (57). In mTOR-driven HCC models, tumor cells also enhance arginine uptake while reducing its conversion to polyamines, thus promoting oncogenic metabolism through the arginine-binding protein RNA binding motif protein 39 (RBM39). Folate-mediated one-carbon (1C) metabolism plays a pivotal role in supplying the building blocks needed for tumor cell proliferation (17, 58–61). In PLC, the expression of key enzymes involved in 1C metabolism is significantly dysregulated (62, 63). The metabolism of serine, glycine, and methionine is closely linked to the generation of 1C units, with glycine-derived 1C units contributing to purine and pyrimidine biosynthesis in HCC, thereby promoting tumor progression through the glycine cleavage system (GCS) (64). Recent studies have also revealed that dietary folate supplementation can enhance tumor development by integrating methionine and 1C metabolism, as observed in DEN/HFD-induced HCC mouse models (65).

### 3.4 Metabolism in iCCA

The metabolism of iCCA shares many features with HCC, including increased glycolytic activity and impaired mitochondrial oxidative phosphorylation. Positron emission tomography using fluorodeoxyglucose (FDG-PET), based on the Warburg effect, has proven valuable in clinical settings for diagnosing, staging, detecting tumor recurrence, guiding treatment, and assessing prognosis (66, 67). Elevated expression of GLUT1 is associated with poor outcomes in both HCC and iCCA (23, 68). The reduced levels of succinic acid and 3-phosphoglycerate in iCCA tumors further indicate the presence of the Warburg effect and increased fatty acid catabolism (54). Pyruvate dehydrogenase kinase 3 (PDK3) expression is significantly higher in iCCA tissues—27 times greater than in benign tissues—and elevated serum PDK3 levels correlate with shorter patient survival (69). Overexpression of PGC-1α enhances OXPHOS in iCCA cells, promoting metastasis (70). HIF-1α transcriptionally upregulates PDK, which suppresses TCA cycle activity while driving the Warburg effect through HIF-1α/PDK signaling (71). Moreover, higher expression levels of PGC-1α or mitochondrial complex II are linked to poorer prognosis and early recurrence in patients with iCCA, suggesting that oxidative mitochondrial metabolism supports CSC populations in iCCA (72). *In vitro* studies on human iCCA cell lines indicate a strong dependence on glutamine. To elucidate the role of glutamine in iCCA, glutamine-independent derivative cell lines (GD cells) were established. These studies showed that hypoxia-induced resistance to cisplatin or gemcitabine was abolished in GD cells, an effect attributed to reduced c-Myc expression, highlighting the role of glutamine metabolism in chemoresistance development in CCA (73).

Unlike in HCC, decreased FASN expression was observed in human iCCA and mouse models. Hydrodynamic injection of AKT/Ras led to the development of both HCC and iCCA lesions in wild-type mice but resulted exclusively in iCCA lesions in FASN knockout mice (74). Furthermore, FASN ablation did not affect iCCA development induced by AKT/Notch intracellular domain 1, indicating that iCCA cells compensate for the absence of FASN by upregulating lipoprotein lipase, CD36, and SLC27A to maintain fatty acid levels. De Gauna et al. (75) observed that rapidly proliferating human iCCA cells depend on lipid and lipoprotein uptake for energy *in vitro* (76), and higher serum levels of CD36 are linked to poorer survival outcomes in patients with iCCA (77). In contrast, high FASN expression correlated with advanced disease stages and lower survival rates in a cohort study involving 155 patients with iCCA from Srinagarind Hospital, Khon Kaen University. Knockdown of FASN in iCCA cells reduced growth, migration, and invasion capabilities *in vitro* (44).

### 3.5 Metabolism in tumor-associated immune cells and HCC

The metabolism of tumor-associated immune cells is crucial in HCC progression, with dysregulated metabolic byproducts from cancer cells significantly impacting immune cells within the TME. Accumulated glucose in the TME enhances CD8+ T cell function by upregulating the costimulatory molecule CD27 through mTOR-FOXM1 signaling, as shown in immunocompetent orthotopic and spontaneous HCC models (78). Meanwhile, lactate, a byproduct of glycolysis, promotes regulatory T cell (Treg) stability and function through lactoylation of Lys72 in MOESIN, whereas reducing lactate levels decreases Treg induction, bolsters anti-tumor immunity, and limits tumor growth (79). Additionally, HCC cells secrete substantial amounts of adenosine, which, in conjunction with granulocyte-macrophage colony-stimulating factor (GM-CSF) from activated tumor-associated macrophages (TAMs), suppresses the immune function of T cells and myeloid cells (80, 81).

Nutrient depletion represents another key mechanism through which tumors suppress immune responses. Glutamine metabolism is vital for both proliferating cancer cells and activated CD8+ T cells. In the TCGA HCC cohort, a higher expression score for genes involved in glutamine metabolism was associated with poorer patient prognosis. *In vitro* co-culture studies demonstrated that glutamine deficiency in the TME induces mitochondrial dysfunction and apoptosis in CD8+ T cells, compromising their tumor-killing capabilities (82). HCC cells compete with CD8+ T cells for glutamine, reducing T cell numbers and impairing their cytolytic function (83). Beyond glutamine, glucose availability is also essential for the metabolic fitness of tumor-infiltrating cytotoxic CD8+ T cells (84). Hypoxia in HCC further contributes to adenosine accumulation and extracellular secretion, exacerbating immune suppression.

Nutritional status within liver cancer tissues also influences macrophage polarization between anti-tumor M1-like macrophages (M1φ) and pro-tumor M2-like macrophages (M2φ), with low ferrous iron levels favoring M2φ polarization (85). The hypoxic environment of HCC drives tumor cells to outcompete macrophages for iron by upregulating the transferrin receptor (TFRC), the primary receptor for transferrin-mediated iron uptake, leading to M2-like TAM polarization *in vitro* (86). Thus, liver cancer cells not only dampen anti-tumor immune activity through nutrient competition but also reshape the metabolic phenotype of immune cells, effectively “stressing” them to sustain tumor growth, thereby facilitating liver cancer development and progression.

Collectively, these metabolic adaptations enable HCC cells to thrive in the TME, driving tumor growth and metastasis. These insights deepen the understanding of HCC’s metabolic complexity and highlight novel potential targets for the diagnosis, treatment, and prognosis of HCC.

### 3.6 Brief summary of metabolism in PLC

Carcinogenic factors disrupt hepatocyte metabolism, initiating HCC, while cancer cells further drive tumor progression through metabolic reprogramming. The reprogramming of glucose, lipid, and amino acid metabolism in HCC cells is intricate and interconnected, with these metabolic pathways mutually reinforcing each other. The interaction between tumor cells and immune and stromal cells creates a complex tumor microenvironment, resulting in various malignant outcomes such as drug resistance, radiotherapy resistance, and tumor recurrence. The similarities and differences in the metabolic reprogramming of HCC and iCCA indicate that therapeutic approaches for these cancers can be informed by each other’s treatment strategies while also requiring differentiation. For instance, Sorafenib has shown some efficacy against both HCC and iCCA *in vitro*, but its clinical effectiveness in iCCA has been limited, restricting its broader application (87). This limitation has also driven research into combination therapies aimed at enhancing Sorafenib’s therapeutic effects on iCCA (88).

Metabolic classification can thus aid in understanding tumor metabolic heterogeneity and guide the development of personalized treatment strategies. Advances in omics technologies have enabled multi-omics approaches that integrate genome, transcriptome, metabolome, and single-cell analyses to elucidate the distinct metabolic landscapes of HCC, leading to the identification of novel prognostic and therapeutic targets. However, these findings urgently require validation through preclinical studies and clinical trials to ensure their effective translation into clinical practice, thereby expanding treatment options for liver cancer.

## 4 Metabolism and MASLD

MASLD is a clinicopathological syndrome characterized by excessive fat accumulation in hepatocytes, excluding alcohol and other established liver-damaging factors, and is closely linked to the development of liver cancer. A 21-year longitudinal study by Allen and colleagues in Olmsted County, Minnesota (1997–2016), compared cancer incidence in adults with MASLD to those without the condition, revealing a nearly threefold higher risk of developing cancer among patients with MASLD. The highest risk was for liver cancer, followed by gastrointestinal and uterine cancers (89). The association between MASLD and cancer risk is even stronger than that observed with obesity alone, with a similar correlation found in a Korean population study (90). Metabolic dysfunction-associated steatohepatitis (MASH), a more severe form of MASLD characterized by inflammation, fat accumulation, and hepatocyte injury, significantly elevates the risk of HCC. In patients with MASH and cirrhosis, the cumulative incidence of HCC ranges from 2.4% to 12.8% over a 7-year period, whereas patients with non-cirrhotic MASLD have a cumulative HCC mortality of 0% to 3% over a span of up to 20 years (91). Approximately 1% of MASH-associated liver cancer cases involve the development of both HCC and CCA. With the global rise in obesity, MASLD/MASH is projected to become the leading cause of HCC in the near future.

Obesity, defined as a body mass index (BMI) of 30 or more, and severe obesity (BMI over 40), significantly elevate the risk of liver cancer. A large U.S. cohort study with 2,162 liver cancer cases reported a 75% increase in liver cancer incidence among obese individuals, with a higher prevalence in men than women (92). Visceral fat, which surrounds abdominal organs such as the liver and pancreas, contributes to fatty liver disease (93, 94), disrupted lipid and glucose homeostasis, and insulin resistance, particularly when metabolized through the portal vein (95). In patients with MASH, the accumulation of toxic saturated fatty acids and ceramides in the liver impairs hepatocyte function. The breakdown of long-chain fatty acids in peroxisomes generates reactive oxygen species (ROS) and toxic lipid intermediates, damaging the mitochondrial respiratory chain, leading to cytochrome c release and apoptosis (96, 97). Furthermore, ROS and oxidative stress can harm the endoplasmic reticulum, activating proteases that cause severe hepatocellular injury and cell death. Insulin resistance and hyperglycemia contribute to the upregulation of insulin and insulin-like growth factor 1 (IGF-1), which stimulate hepatocyte proliferation and inhibit apoptosis through activation of the PI3K and MAPK signaling pathways (98).

**Figure 3:**
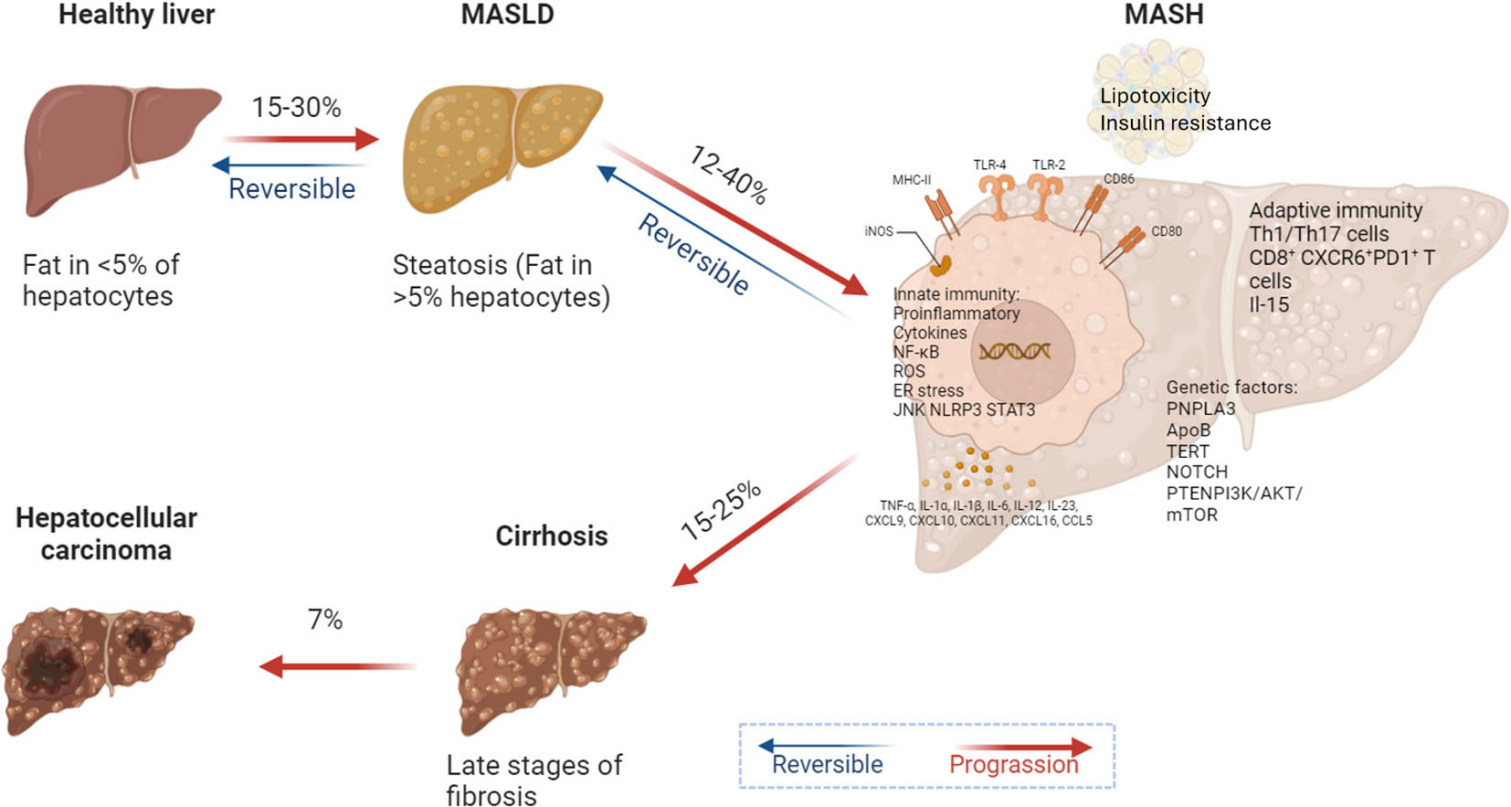
Metabolic reprogramming drives MASLD progression. In MASLD and its more advanced form, MASH, lipid toxicity and insulin resistance create a microenvironment conducive to HCC development. Activation of both innate and adaptive immune responses, along with cytokine release and CD8+ T cell infiltration, promotes the expression of genes associated with cell proliferation, migration, and survival. Key regulatory molecules such as SOCS3 and PTEN are downregulated, failing to inhibit oncogenic signaling pathways in liver cancer. A high-fat diet and excessive carbohydrate intake exacerbate pro-inflammatory cytokine profiles and increase DNL in the liver, promoting lipid peroxidation and ultimately leading to cirrhosis and liver cancer.

Dysregulation of signaling pathways in MASLD is a key factor in liver cancer development. Notch signaling, implicated in MASH-associated fibrosis, plays a significant role in MASLD progression (99–101). Zhu et al. demonstrated that MASH-induced sustained Notch activation promotes tumorigenesis even without genomic driver mutations in genes like CTNNB1 or Notch pathway components (102). Teresa Auguet and colleagues further explored the correlation between Notch signaling and MASLD severity, finding that the expression of Notch proteins and ligands was positively associated with genes involved in hepatic lipid metabolism and Toll-like receptor expression (103). Additional pathways, including TNFα, IL6, and androgen-mediated signaling, contribute to increased hepatic cell turnover. The combined effects of ROS, oxidative stress, and chronic inflammation increase the likelihood of DNA mutations, with genetic instability in patients with MASH estimated to be 10 to 20 times higher than in those with MASLD. Mutations in genes such as PNPLA3, TM6SF2, MBOAT7, GCKR, HSD17β13, and MARC1 are commonly found in patients with MASH (98, 104). Evidence suggests that the development of HCC driven by MASH is a multifactorial process involving disrupted lipid metabolism, mitochondrial dysfunction, oxidative stress, inflammatory signaling, and even the influence of the gut microbiota (**Figure 3**).

## 5 Therapeutic strategies targeting metabolic reprogramming in PLC

Metabolic reprogramming is a key target in cancer therapy, leading to the development and FDA approval of various drugs targeting specific metabolites or metabolic pathways for PLC treatment. *In vitro*, preclinical, and clinical studies have explored metabolic targets for PLC therapy (**Table 1**).

**Table 1:** Clinical trials on metabolic targets in PLC.

Metabolic Pathway	Metabolic Target	Compounds	Cancer Type	Clinical Trials or Approved Drugs	References
Glycolysis	Glut1	Aspirin	HCC	FDA approved drug	(122, 123)
	mTOR	Everolimus	CCA	FDA approved cancer drug	(124)
	PDK	Dichloroacetate	Recurrent and/or metastatic solid tumors	NCT00566410	(125)
	MCT1	AZD-3965	Advanced cancer	NCT01791595	(126)
TCA cycle	Mitochondrial complex I	Metformin	HCC recurrence after hepatic resection, Advanced solid tumors	NCT03184493	(127, 128)
				NCT02145559	
				NCT04033107	
				NCT02672488	
				NCT02496741	
	IDH1/2	AG120(Ivosidenib)	Previously treated patients with nonresectable or metastatic CCA	NCT02989857	(129–131)
				NCT06081829	
				NCT05876754	
				NCT04088188	
		AG120(Ivosidenib) combined with Nivolumab/Ipilimumab	Advanced solid tumors (including CCA)	NCT05921760	
				NCT02073994	
				NCT05921760	
				NCT02073994	
		Metformin + chloroquine	CCA	CCA	
Lipid metabolism	FASN	TVB2640	MASLD	NCT03938246	(111, 112, 132)
	HMG-CoA reductase	Pravastatin	HCC	NCT01075555	(133, 134)
				NCT01357486	
				NCT01418729	
				NCT01903694	
		Atorvastatin	HCC recurrence after curative treatment	NCT03024684	
		Simvastatin	HCC in patients with cirrhosis	NCT02968810	
		Statin	HCC recurrence after liver transplantation	NCT03490461	
	SPHK2	ABC294640	CCA, CCA, advanced solid tumor	NCT03377179	(114)
				NCT01488513	
				NCT02939807	
Amino acid metabolism	Glutaminase	CB-839	HCC	NCT02071862	(116)
	Glutamine	DRP-104 combined with Durvalumab	Advanced fibrolamellar HCC	NCT06027086	

### 5.1 Targeting the glucose metabolism

Metformin, commonly used for glycemic control in patients with diabetes, has shown significant anti-liver cancer effects. *In vitro* studies demonstrate that metformin inhibits HCC cell proliferation, migration, and invasion, reduces tumor growth in xenograft mouse models, and extends survival (105, 106). Clinical trials indicate that metformin therapy can lower the incidence of type 2 diabetes (107). In iCCA, metformin reduces cancer stem cell (CSC)-associated marker expression and suppresses tumor growth in mouse models (72). Currently, three ongoing clinical trials are investigating metformin’s effects in HCC (NCT03184493, NCT04033107, NCT04114136), and a Phase IB/II trial is underway for iCCA (NCT02496741). Approximately 20% of cholangiocarcinomas harbor IDH1 and IDH2 mutations; ivosidenib has been approved for treating IDH1-mutant CCA.

Targeted therapies against GLUT1 have been evaluated for HCC and CCA treatment. Local administration of the Glut1 inhibitor BAY-876 effectively reduces glucose uptake, proliferation, and epithelial-mesenchymal transition in mouse HCC models. The mTOR inhibitor everolimus impedes glucose uptake and tumor angiogenesis by downregulating HIF-1α expression. Additionally, resveratrol enhances the efficacy of sorafenib in HCC by suppressing HK2 expression (108). Similarly, 2-deoxy-D-glucose (2-DG), a glucose analog, sensitizes mouse liver tumors to sorafenib by targeting HK2 activity (109).

### 5.2 Targeting the lipid metabolism

Elevated lipid synthesis is a hallmark of PLC, making it a major focus of metabolic therapies. Sterol regulatory element-binding protein 1c (SREBP-1c) is a key regulator of lipogenesis; its inhibition induces cell cycle arrest and apoptosis in HCC, whereas overexpression promotes tumor proliferation (47). Fatostatin, a small molecule that inhibits SREBP activation, reduces body weight, blood glucose, and liver fat accumulation in obese ob/ob mice (110) and suppresses prostate cancer growth and metastasis in mouse models (47, 111). Lipastatin derivatives, such as FGH10019 (compound 24), have shown promise due to their high water solubility and membrane permeability, positioning them as seed molecules for developing anti-SREBP drugs (112). FASN inhibitors, such as TVB3664, prevent HCC development driven by PTEN/c-MET or AKT/NRAS mutations and enhance the efficacy of sorafenib or cabozantinib in c-Myc-driven HCC (113).

Conserved lipid kinases SPHK1 and SPHK2 produce sphingosine-1-phosphate (S1P), which supports cancer cell survival. In a Phase I clinical trial for advanced solid tumors, a partial response was observed in a patient with advanced CCA treated with the S1P inhibitor ABC294640. Ongoing studies include a Phase I/IIA trial in iCCA (NCT03377179) and a Phase II trial in HCC (NCT02939807) (114).

### 5.3 Targeting the amino acid metabolism

Crisantaspase, an anti-leukemia agent that depletes cellular glutamine, effectively reduced glutamine availability and inhibited HCC growth in xenograft mouse models when combined with methionine-L-sulfoxide (115). Glutaminase 1 (GLS1) facilitates the conversion of glutamine to glutamate, and co-treatment with the GLS1 inhibitor CB-839 and the glutamine transporter inhibitor V-9302 induced apoptosis in mouse liver cancer cells and suppressed HCC xenografts (116). Hydroxychloroquine, an autophagy inhibitor approved for rheumatoid arthritis, demonstrated superior efficacy in a Phase II clinical trial for advanced HCC, with a combination of sorafenib and hydroxychloroquine achieving a response rate of 25%, compared to 2% with sorafenib alone (NCT03037437). Ongoing trials are evaluating the combination of trametinib (a MEK inhibitor) and hydroxychloroquine in KRAS mutation-resistant CCA (NCT04566133).

### 5.4 Targeting the metabolism of tumor-associated immune cells

The interaction between cancer cells and immune cells within the TME often limits the effectiveness of immunotherapy. The integration of immune and metabolic therapies has emerged as a promising strategy. In a low-glucose TME, tumor cells produce lactic acid through glycolysis, which is absorbed by Tregs *via* monocarboxylate transporter 1 (MCT1), facilitating NFAT1 translocation into the nucleus and upregulating PD-1 expression. In contrast, effector T cells downregulate PD-1 expression, leading to immune escape by activated Tregs expressing PD-1, which contributes to therapeutic failure (117). Combining the MCT1 inhibitor AZD3965 with anti-PD-1 therapy offers a novel approach to overcome this challenge.

IFNα can inhibit HIF1α signaling to decrease glucose consumption by tumor cells, resulting in a higher-glucose TME. This increased glucose availability stimulates CD27 expression on CD8+ T cells *via* mTOR-FOXM1 signaling, thereby enhancing cytotoxic T cell activity in immunocompetent and spontaneous HCC models. Consequently, IFNα and anti-PD-1 combination therapy represents a promising strategy for HCC treatment (78). Additionally, ongoing clinical trials are investigating AG120 (ivosidenib) in combination with nivolumab/ipilimumab and the glutamate analog DRP-104 with durvalumab, further exploring the potential of combined metabolic and immune-targeted therapies.

### 5.5 Targeting the MASLD

Given the association between obesity, MASLD, and increased HCC risk, strategies such as caloric restriction, weight loss, and diabetes management are essential for HCC prevention. Short-term fasting (24–72 hours) or intermittent fasting has been investigated as a potential approach to prevent or treat HCC. The underlying theory is that during energy deprivation, healthy cells halt growth and enter maintenance and repair modes, which protects them from chemotherapy or radiation-induced damage. In contrast, cancer cells persist in their growth despite nutrient scarcity, making them more vulnerable to anti-cancer therapies (118). In various cancer models, short-term fasting has been shown to reduce chemotherapy-related toxicity, enhance the efficacy of chemotherapy and radiation, and improve survival rates in animal studies. In a DEN-induced liver cancer rat model, intermittent fasting significantly decreased the number and size of precancerous nodules after 52 weeks of observation (119). Another study demonstrated that short-term fasting led to a temporary reduction in liver weight and regression of precancerous liver lesions by decreasing hepatocyte volume and proliferation and inducing apoptosis (120). However, fasting followed by refeeding can paradoxically accelerate hepatocarcinogenesis, potentially due to compensatory cell proliferation. For instance, adding three cycles of 3-day fasting to a tumor-promoting regimen doubled the incidence of HCC compared to control rats. Similarly, refeeding after fasting hastened HCC development in response to a sublethal dose of DEN (20 mg/kg), suggesting that fasting followed by refeeding promotes hepatocyte turnover, thereby increasing HCC risk, which contrasts with the effects seen in other organs (121).

Overall, research on metabolic reprogramming in cancer is gradually uncovering the ways tumor cells exploit metabolic pathways to support survival and proliferation, providing a scientific foundation for metabolic-based therapeutic strategies. From mitochondrial function and the roles of specific metabolic enzymes to the interplay between metabolism and the immune system, these studies collectively outline a broad landscape for future cancer treatments. The findings suggest that metabolic therapy could become a key component of a comprehensive cancer treatment strategy.

## 6 Conclusions and prospects

In summary, metabolic reprogramming is pivotal in the pathogenesis of PLC, characterized by increased glycolysis, disrupted lipid metabolism, and altered amino acid pathways, with intricate interactions involving the TME and immune system. Metabolic dysfunction associated with MASLD heightens liver cancer risk, while metabolite exchange between tumor and immune cells facilitates immune evasion and tumor progression.

However, as with other malignancies, treating liver cancer through metabolic intervention presents significant challenges. Firstly, since metabolism is essential for the survival of all cells, therapies must selectively target cancer cells while preserving normal cellular function. Secondly, the inherent adaptability and heterogeneity of tumor metabolism make liver cancer prone to developing drug resistance. Thus, a precise understanding of tumor subtype-specific metabolic traits and employing combination therapies to minimize resistance are critical.

Future liver cancer treatment strategies must evolve to address the complexities of metabolic reprogramming. Integrating metabolomics, genomics, and proteomics will facilitate the identification of more specific and effective therapeutic targets. Furthermore, combining metabolic therapies with immunotherapy is anticipated to be a major research focus, aiming to enhance immunotherapy responses and overcome resistance by modulating the metabolic state of the TME. Continuous investigation into the metabolic regulatory networks of liver cancer will support more accurate disease classification, innovative metabolic therapies, and improved clinical outcomes.
